# Characteristics of antifungal utilization for hospitalized children in the United States

**DOI:** 10.1017/ash.2022.338

**Published:** 2022-12-02

**Authors:** Lourdes Eguiguren, Brian R. Lee, Jason G. Newland, Matthew P. Kronman, Adam L. Hersh, Jeffrey S. Gerber, Grace M. Lee, Hayden T. Schwenk

**Affiliations:** 1 Division of Pediatric Infectious Diseases, Department of Pediatrics, University of Nebraska Medical Center, Omaha, Nebraska; 2 Division of Health Services and Outcomes Research, Department of Pediatrics, Children’s Mercy Kansas City, Kansas City, Missouri; 3 Division of Infectious Diseases, Department of Pediatrics, Washington University in St Louis, St Louis, Missouri; 4 Division of Pediatric Infectious Diseases, Department of Pediatrics, University of Washington, Seattle, Washington; 5 Division of Pediatric Infectious Diseases, Department of Pediatrics, University of Utah, Salt Lake City, Utah; 6 Division of Pediatric Infectious Diseases, Department of Pediatrics, Children’s Hospital of Philadelphia, Philadelphia, Pennsylvania; 7 Division of Pediatric Infectious Diseases, Department of Pediatrics, Stanford University, Stanford, California

## Abstract

**Objective:**

To characterize antifungal prescribing patterns, including the indication for antifungal use, in hospitalized children across the United States.

**Design:**

We analyzed antifungal prescribing data from 32 hospitals that participated in the SHARPS Antibiotic Resistance, Prescribing, and Efficacy among Children (SHARPEC) study, a cross-sectional point-prevalence survey conducted between June 2016 and December 2017.

**Methods:**

Inpatients aged <18 years with an active systemic antifungal order were included in the analysis. We classified antifungal prescribing by indication (ie, prophylaxis, empiric, targeted), and we compared the proportion of patients in each category based on patient and antifungal characteristics.

**Results:**

Among 34,927 surveyed patients, 2,095 (6%) received at least 1 systemic antifungal and there were 2,207 antifungal prescriptions. Most patients had an underlying oncology or bone marrow transplant diagnosis (57%) or were premature (13%). The most prescribed antifungal was fluconazole (48%) and the most common indication for antifungal use was prophylaxis (64%). Of 2,095 patients receiving antifungals, 79 (4%) were prescribed >1 antifungal, most often as targeted therapy (48%). The antifungal prescribing rate ranged from 13.6 to 131.2 antifungals per 1,000 patients across hospitals (*P* < .001).

**Conclusions:**

Most antifungal use in hospitalized children was for prophylaxis, and the rate of antifungal prescribing varied significantly across hospitals. Potential targets for antifungal stewardship efforts include high-risk, high-utilization populations, such as oncology and bone marrow transplant patients, and specific patterns of utilization, including prophylactic and combination antifungal therapy.

Invasive fungal disease (IFD) poses a serious risk to critically ill, premature, and immunocompromised patients. Given the high rates of morbidity and mortality related to IFD, current clinical guidelines recommend the use of prophylactic antifungals and the early initiation of empiric antifungal therapy for certain high-risk populations.^1–3^ Consequently, the use of systemic antifungals in hospitalized children has increased, particularly in immunocompromised and neonatal populations.^4^ Existing data suggest that despite the availability of consensus guidelines for the prophylaxis and treatment of common fungal infections, there is significant variability in antifungal prescribing across institutions and geographic regions.^5,6^ The reasons behind these differences in practice remain unclear; however, poor adherence to guidelines may be a sign of overuse or inappropriate use. It has been estimated that ∼50% of the systemic antifungals prescribed are inappropriate (eg, incorrect dose, incorrect frequency, incorrect route, etc) and that 16% are unnecessary.^7^ Suboptimal antifungal use can result in treatment failure, adverse drug events, development of antifungal resistance, and increased healthcare costs.^8–11^

Antimicrobial stewardship is an important tool to decrease the unnecessary and suboptimal use of antimicrobials. However, most current efforts have targeted antibiotic use, whereas antifungal stewardship has been relatively overlooked.^10,12,13^ One possible barrier to the development and implementation of antifungal stewardship interventions is a lack of understanding of antifungal prescribing patterns in pediatric patients. In general, antifungals are mostly used for the prevention of IFD in high-risk populations (ie, prophylaxis), treatment of possible or probable IFD (ie, empiric therapy), or treatment of proven IFD (ie, targeted therapy). Studies describing the indications for antifungal use, including whether the antifungal therapy is prophylactic, empiric, or targeted are limited. Understanding the indications for use can help direct and prioritize future stewardship efforts aimed at optimizing prophylaxis for the prevention of infection, optimizing empiric therapy to avoid unnecessary antifungal exposure, and optimizing directed therapy to ensure the best treatment outcomes. In this study, we characterized antifungal prescribing patterns, including the indication for use, in hospitalized children across the United States.

## Methods

### Study design

We analyzed antifungal prescribing data from 32 children’s hospitals that participated in the SHARPS Antibiotic Resistance, Prescribing, and Efficacy among Children (SHARPEC) study.^14,15^ The SHARPEC study was a prospective, cross-sectional point prevalence survey and our analysis included data collected between June 2016 and December 2017. Participating hospitals selected a survey date within a specified 3-week period each calendar quarter during the study period. Each hospital collected data on all active antimicrobial prescriptions on the selected day of the survey. The study was approved by the institutional review board (IRB) at Children’s Mercy Hospital, which was the coordinating site, and at individual sites, as needed, per institutional IRB policies.

### Study population

Data from all hospitalized patients under 18 years of age, admitted at 08:00 on the selected day of each point prevalence survey, with an active systemic or inhaled antifungal prescription, were included in this analysis. Topical antifungals and those with minimal enteral absorption (eg, nystatin) were excluded from the analysis.

### Data collection

Data for the SHARPEC study were collected by the antimicrobial stewardship program physician or pharmacist from each hospital by individual chart review at each site. A standardized data collection form was used to record demographic and antimicrobial prescribing information, including patient age, sex, underlying diagnoses, hospital ward, hospital ward activity (ie, medical, surgical, intensive care), antifungal agent, antifungal route of administration, and antifungal indication. Deidentified data from each site were entered into an online REDCap database developed and maintained by Children’s Mercy Hospital.

The antimicrobial stewardship program (ASP) physician or pharmacist selected the patient’s underlying diagnosis from a list of 19 prespecified categories.^15^ Up to 3 underlying diagnoses could be selected for each patient. We used the following diagnosis categories: oncology or bone marrow transplant (Onc-BMT), prematurity, chronic lung disease, gastroenterological disease, congenital heart disease, hematology or sickle-cell disease, surgical disease, and solid-organ transplant. The remaining diagnoses were recategorized as “other.” Hospital wards were selected from the following options: general pediatric medical ward, hematology-oncology, cardiology, transplant (bone marrow and solid organ), other special pediatric medical ward, pediatric surgical ward, pediatric intensive care unit (PICU), neonatal ICU (NICU), cardiac ICU, and other. Routes of administration included intravenous, enteral, and inhaled.

Based on individual patient chart review, ASP teams were asked to assess whether the indication for treatment was for a community-acquired infection, healthcare-associated infection, prophylaxis, not used for prophylaxis or treatment purposes, or unknown, and whether the treatment was empiric or targeted. Using this information, we reclassified each antifungal prescription as prophylaxis, empiric, or targeted therapy (Supplementary Table 1). All antifungal prescriptions with an indication of prophylaxis were categorized by the study team as prophylaxis. All other indications were considered empiric or targeted therapy. Antifungal prescriptions were categorized as empiric therapy when the following options were selected: empiric without culture(s) performed; empiric but culture and susceptibility testing pending; or, empiric without positive cultures. Because obtaining antifungal susceptibilities is not always feasible, prescriptions were considered targeted therapy when the following options were selected: empiric and pathogen identified or targeted and pathogen and antimicrobial resistance confirmed.

### Data analysis

Antifungal prescribing data from all hospitals and quarters were pooled for analysis. We used descriptive statistics to characterize demographic and antifungal prescribing data. For patient age, we used the following categories: 0–28 days (neonate), 29 days–1 year (infant), 1–2 years (toddler), 3–5 years (early childhood), 6–11 years (middle childhood), and 12–17 years (adolescence).^16^ Patient age was reported as the proportion of patients in each age category. We classified antifungal prescribing by indication (prophylaxis, empiric, targeted), and we identified the percentage of antifungal prescriptions in each category.

We compared clinical and antifungal prescribing characteristics including age, sex, hospital ward, antifungal class, and use of antifungal combination therapy based on antifungal indication. We classified antifungal agents into the following categories: fluconazole; other azole (voriconazole, posaconazole, isavuconazole, itraconazole, ketoconazole); echinocandin (micafungin, caspofungin, anidulafungin); polyene (conventional and liposomal amphotericin); and, other (flucytosine, griseofulvin, terbinafine). We defined combination therapy as patients receiving 2 or more systemic antifungals and did not include inhaled antifungals in this analysis given their low systemic absorption. We determined the percentage of patients who were prescribed combination therapy and reported the proportion of patients prescribed different drug combinations by antifungal class and antifungal agent.

We used the Fisher exact test and the Pearson χ^2^ test to compare categorical variables, as appropriate, for comparisons. Numerical variables that that did not follow a normal distribution were expressed as medians and interquartile ranges (IQRs). Multivariable Poisson regression models were used to assess variability in antifungal use across hospitals. The SHARPEC hospital was assumed as a fixed effect, while adjusting for hypothesized confounders (ie, hospital ward, hospital ward activity, whether the treated patient had underlying chronic conditions) and offsetting the patient census counts. Postestimation marginal effects were performed to calculate regression-adjusted antifungal prescription rates (antifungal prescriptions per 1,000 patients). The statistical analyses were performed with JMP version 14.1 software and SAS version 9.4 software (SAS Institute, Cary, NC).

## Results

### Hospital characteristics

In total, 32 hospitals participated in the study by contributing data from at least 1 survey cycle; 16 hospitals contributed data for all 6 cycles. There was no difference in antifungal prescriptions per 1,000 patients (137.4 vs 140.1; *P* = .837) or bed count (307 vs 295; *P* = .751) between hospitals reporting data in all 6 quarters and the remainder of hospitals. Participating hospitals were located in 22 unique states within the United States, representing all 4 Census regions. The median hospital bed count was 302 (IQR, 146–366). Also, 31 hospitals included data from a NICU ward, 30 hospitals provided hematology-oncology ward data, and 9 hospitals provided data from a transplant ward.

### Patient characteristics

During the study period, 2,095 (6%) of 34,927 surveyed patients received at least 1 antifungal medication, and there were 2,207 antifungal prescriptions. The characteristics of the patients receiving antifungal drugs are described in Table 1. The median age among patients receiving antifungals was 4 years (IQR, 0–11) and 25% of patients were aged <1 year. Among patients admitted to the NICU who were receiving an antifungal, the median gestational age at birth was 26 weeks (IQR, 24–30), the median birth weight was 800 g (IQR, 640–1,400), and the median weight at the time of antifungal use was 1500 g (IQR, 900–3,100). The most frequent underlying diagnoses were Onc-BMT in 1,200 (57%) of 2,095 patients and prematurity in 268 patients (13%). Most children with antifungal prescriptions were admitted to a hematology-oncology ward (46%) or NICU (14%).

**Table 1.** Demographic and Clinical Characteristics of Hospitalized Patients Receiving Systemic Antifungals

Patient Characteristic	Total Patients (N = 2,095), No. (%)
**Age groups**	
0–28 d	141 (7)
29 d–1 y	390 (18)
1–2 y	316 (15)
3–5 y	290 (14)
6–11 y	469 (22)
12–17 y	489 (24)
**Sex^a^**	
Male	933 (45)
Female	1154 (55)
**Diagnosis**	
Oncology or BMT	1200 (57)
Prematurity	268 (13)
Chronic lung disease	233 (11)
Gastroenterological disease	205 (10)
Congenital heart disease	154 (7)
Hematology/Sickle-cell disease	150 (7)
Surgical disease	125 (6)
Solid-organ transplant	100 (5)
Other	652 (31)
**Hospital ward^b^**	
Hematology-Oncology	924 (46)
NICU	287 (14)
PICU	261 (13)
Transplant (BMT or solid organ)	182 (9)
General pediatric medical ward	146 (8)
Cardiac intensive care unit	89 (4)
Pediatric surgical ward	47 (2)
Other^c^	89 (4)

Note. BMT, bone marrow transplant; NICU, neonatal intensive care unit; PICU, pediatric ICU.

a Missing N = 8.

b Missing N = 70.

c Includes cardiology, other special medical ward, and other.

### Antifungal prescribing characteristics

The characteristics of antifungal prescribing among hospitalized pediatric patients are shown in Table 2. The most prescribed antifungal was fluconazole, accounting for 48% of the prescriptions, followed by echinocandins (23%) and other azoles (20%). Among 2,207 antifungal prescriptions, 1,420(64%) were for prophylaxis, 429 (20%) were for empiric therapy, 308 (14%) were for targeted therapy, and 50 (2%) did not have a documented indication. Also, 1,296 (59%) of antifungal prescriptions were administered parenterally, and 624 (48%) of those administered parenterally were azole antifungals (Supplementary Table 2). Among 2,095 patients 79 (4%) received >1 systemic antifungal, accounting for 177 (8%) of 2,207 antifungal prescriptions. As shown in Table 3, among patients receiving combination antifungal therapy, the most prescribed drug combinations were polyene-azole (43%), echinocandin-azole (36%), and polyene-echinocandin (13%).

**Table 2.** Characteristics of Antifungal Prescribing Among Hospitalized Children

Antifungal Prescribing Characteristic	Antifungal Prescriptions (N = 2,207), No. (%)
**Antifungal agents**	
Fluconazole	1,051 (48)
Echinocandin	517 (23)
Other azole	442 (20)
Polyene	184 (8)
Other^a^	13 (1)
Indication	
Prophylaxis	1,420 (64)
Empiric	429 (20)
Targeted	308 (14)
Unknown	50 (2)
Route^b^	
Intravenous	1,296 (59)
Oral	877 (40)
Inhaled	29 (1)
Combination therapy	
Yes	177 (8)

a Other azole: voriconazole, posaconazole, isavuconazole, itraconazole, ketoconazole.

b Missing N = 5.

**Table 3.** Patients Receiving Combination Antifungal Therapy by Antifungal Class Combination and Antifungal Agent Combinations

Antifungal Class Combinations	Patients (N = 79), No. (%)	Antifungal Agent Combinations	Patients, No.
Polyene-azole	34 (43)	AmB-voriconazole	14
		AmB-posaconazole	11
		AmB-fluconazole	8
		AmB-itraconazole	1
Echinocandin-azole	29 (36)	Echinocandin-voriconazole	16
		Echinocandin-posaconazole	7
		Echinocandin-fluconazole	5
		Echinocandin-isavuconazole	1
Polyene-echinocandin	10 (13)	AmB-micafungin	10
Other combinations	6 (8)	AmB-flucytosine	1
		Posaconazole-terbinafine	1
		Voriconazole-flucytosine	1
		AmB-posaconazole-caspofungin	1
		AmB-posaconazole-terbinafine	1
		AmB-voriconazole-terbinafine	1

Note. AmB, amphotericin B.

### Indications for antifungal use

**Fig. 1.**
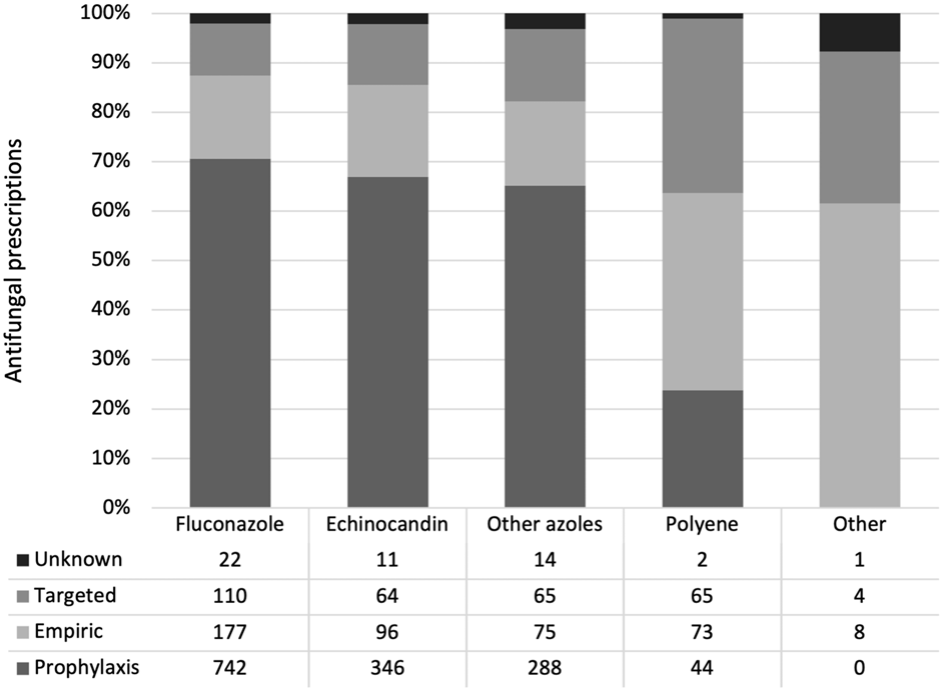
Comparison of antifungal indications by antifungal class.

**Fig. 2.**
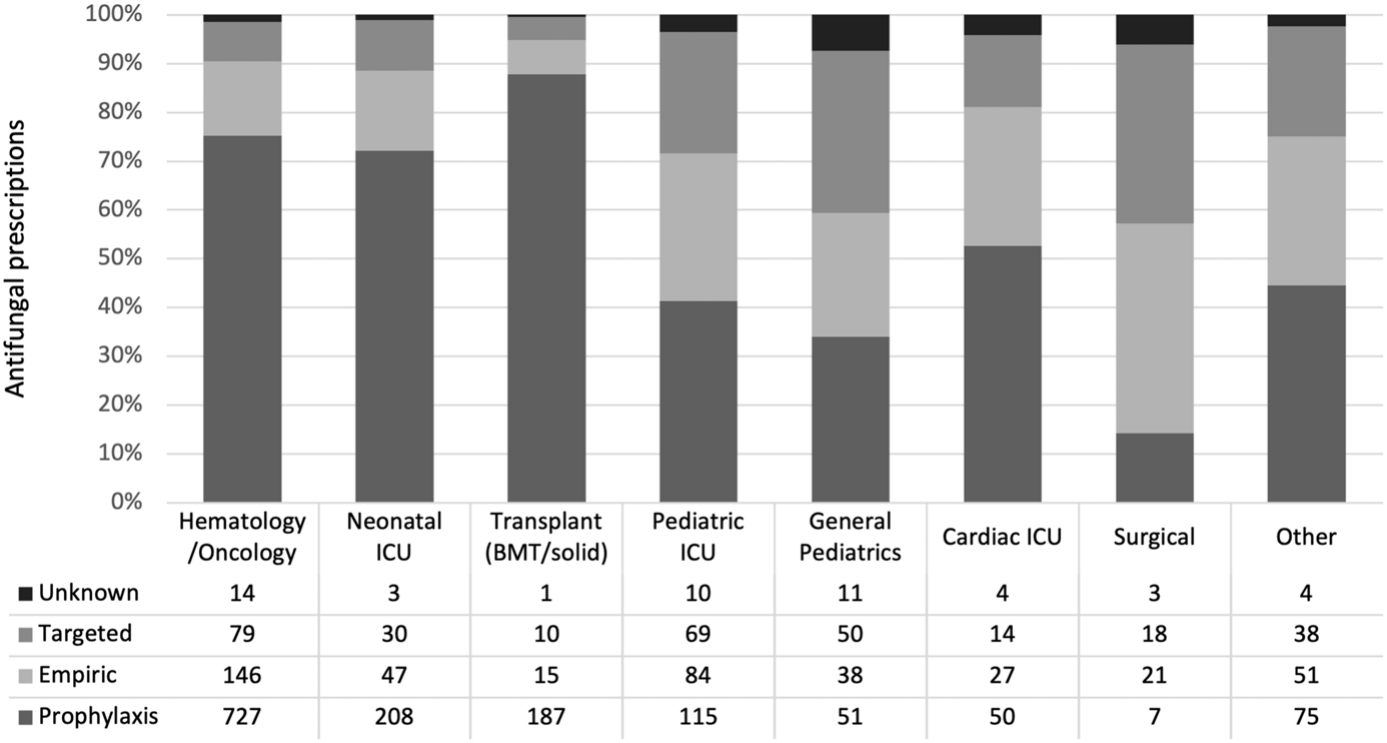
Comparison of antifungal indications by hospital ward. Note. ICU, intensive care unit; BMT, bone marrow transplant.

There were significant differences between prophylactic, empiric, and targeted antifungal use, based on clinical and antifungal prescribing characteristics. Patients receiving antifungals for prophylaxis were younger (median, 4.0 years; IQR, 0–11) compared to those receiving empiric (median, 6.0 years; IQR, 1–12) or targeted (median, 7.0 years; IQR, 1–13) antifungal therapy. The majority of echinocandin (67%), fluconazole (70%), and other azole (65%) prescription use was for prophylaxis, whereas polyenes were mostly used for empiric and targeted therapy (40% and 35% respectively) (Fig. 1). The most used antifungal for prophylaxis, empiric, and targeted therapy was fluconazole (52%, 41%, and 36%, respectively). More than two-thirds of the antifungal prescriptions in the hematology-oncology, NICU, and transplant wards were for prophylaxis (Fig. 2). Nearly one-third of the antifungals used for targeted therapy were prescribed as combination therapy, compared to 12% of those used for empiric therapy, and 3% of those used for prophylaxis.

### Antifungal utilization variability

**Fig. 3.**
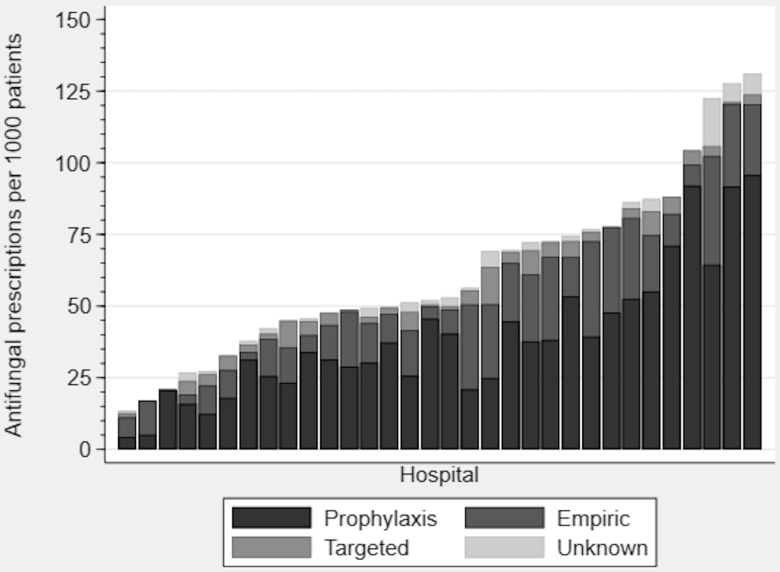
Variation in antifungal utilization and indications by hospital.

Figure 3 shows variability in antifungal utilization and indication for use across participating hospitals. Antifungal prescribing rates ranged from 13.6 to 131.2 antifungal prescriptions per 1,000 patients (median, 50.3; IQR, 42.6–76.7; *P* < .001). The SHARPEC hospital was significantly associated with antifungal use, after accounting for hospital ward and patient factors (likelihood ratio χ^2^ *P* < .0001).

## Discussion

Using this point-prevalence survey of nearly 35,000 patients seen at 32 hospitals over 18 months, we identified several prescribing patterns that can be used to inform antifungal stewardship targets in hospitalized neonates and children. Overall, 6% of hospitalized children were prescribed a systemic antifungal, most commonly fluconazole. Most of the antifungal use was for prophylaxis, mostly for patients with an underlying oncologic diagnosis and premature neonates. Combination antifungal therapy was uncommon but was used for nearly one-third of patients receiving targeted treatment. Importantly, there was also significant variability in antifungal use between participating hospitals, signaling potential opportunities to optimize the use of these antimicrobials.

Fluconazole is the most commonly prescribed antifungal in children^4,5,17^; however, recent trends have shown that the use of other antifungals, including echinocandins and mold active-azoles, is increasing.^18–20^ In a study describing antifungal use in children’s hospitals in the United States between 2006 to 2012, ∼70% of patients receiving a systemic antifungal were prescribed fluconazole.^18^ Similar to our data, in a more recent study from 2015, fluconazole prescriptions were lower than previously reported, accounting for just 50% of the total antifungal prescriptions in pediatric patients.^17^ The exact reasons behind this shift in fluconazole prescribing are unknown. Although a rise in the incidence of IFD in children requiring treatment with broader spectrum antifungals is a possibility, studies have shown that rates of invasive candidiasis and aspergillosis in children have decreased or remained relatively stable over time.^21,22^ Alternatively, the decrease in fluconazole use may reflect recent guidelines recommending the use of an echinocandin as initial therapy for patients with candidemia.^23^ Because the main indication for antifungal use in children is prophylaxis, it is also possible that this shift is the result of increasing use of echinocandins and mold active-azoles for prophylaxis in high-risk oncology patients.^24^ Given the increase in use of broad-spectrum antifungals among hospitalized children, potential negative sequelae, including the emergence of more resistant fungal pathogens, should be the focus of future studies.

Previous European studies describing antifungal use in hospitalized children and neonates have shown that most antifungal use in this population is for the prevention of IFD.^4,25^ Similar data in pediatric hospitals in the United Stated are lacking.^17,18^ In our study, the main indication for antifungal use in children is prophylaxis, followed by empiric and targeted therapy. This finding is similar to recently published data that identified prophylaxis as the second most common indication for antibiotic prescribing.^15^ The high proportion of antifungal prophylaxis may be related to the development of clinical guidelines recommending the use of prophylactic therapy to prevent IFD in high-risk populations, such as extremely low-birthweight neonates^23,26^ and children receiving chemotherapy or undergoing hematopoietic stem-cell transplant.^24,27,28^

Consistent with these guidelines, our study showed that more than two-thirds of the antifungal use in the hematology-oncology unit, the NICU, and the transplant unit was for prophylaxis, whereas empiric and targeted therapy was predominant in other hospital wards. Although the use of antifungal prophylaxis has the potential to decrease the incidence of IFD^29^ and improve outcomes,^30^ if used inappropriately or overused there is potential for toxicities, emergence of antifungal resistance, and increased healthcare costs. Given that the number of children at risk for IFD, including those born prematurely or diagnosed with cancer, continues to rise,^31,32^ further studies are urgently needed to evaluate the most effective approach (ie, antifungal class, duration, etc) to antifungal prophylaxis in vulnerable populations. Antimicrobial stewardship programs could play a key role in evaluating the appropriateness of antifungal prophylaxis and optimizing these drugs to maximize potential benefits and minimize toxicities.

Although combination therapy was only used in 4% of the patients receiving antifungals, we found that almost one-third of antifungal prescriptions for targeted therapy included a combination of 2 or more antifungals. Studies have shown mixed results regarding the effectiveness of using combination therapy to treat IFD.^33,34^ Although in vitro data suggest synergistic activity of some antifungals when used in combination,^35,36^ the clinical benefit is unknown. Prior studies have found that the use of combination therapy in the treatment of IFD does not improve outcomes and is associated with greater frequency of adverse events.^33,37^ Except for certain specific indications, most clinical guidelines recommend the use of monotherapy for the treatment of IFD, including candidiasis and aspergillosis.^23,38^ Despite these recommendations, we found that combination therapy for targeted treatment is relatively common. The use of antifungal combination therapy in children may represent an important stewardship target, but additional studies evaluating the risks and benefits of specific antifungal combinations in children with IFD are needed.

Similar to previous studies of antifungal use,^5,17^ we detected significant variability in antifungal prescribing across participating hospitals. The reasons behind this variability are not known. Prior studies have demonstrated similar variability in antibiotic prescribing, even after accounting for differences in hospital- and patient-level characteristics.^39^ The differences in antifungal prescribing may reflect the incidence of IFD across hospitals, whereby hospitals with higher rates of IFD would be expected to have higher antifungal use. However, our data show that antifungal prescribing is mainly driven by prophylactic and empiric, rather than targeted, therapy. There may be variability in the proportion of high-risk patients requiring prophylaxis across the surveyed hospitals. Alternatively, this variability may represent the lack of a single approach to antifungal prophylaxis in at-risk children. Regardless of the cause, the variability in antifungal prescribing likely signals an opportunity to optimize the use of these agents. Future studies should evaluate whether there is any relationship between the rate of antibiotic and antifungal prescribing at the hospital level, the appropriateness of antifungal use in children, and whether the observed differences in antifungal prescribing are associated with differences in patient outcomes.

This study had several limitations. In this point-prevalence study, we only collected data on 6 single days; therefore, we were not able to determine the duration of antifungal use for each patient or compare the days of therapy between institutions. However, point-prevalence surveys are an important and validated tool to describe antimicrobial prescribing patterns, including indications for use.^15^ Because this was a multicenter study, such an approach facilitates the standardization of data collection and comparison between hospitals and over time. We were not able to determine the appropriateness of antifungal use. Future iterations of similar point prevalence surveys may consider focusing on appropriateness of antifungal use. The indication for antifungal use was determined by the respondent at each institution, and we did not collect microbiological data, which may have led to some subjectivity in differentiating between prophylactic, empiric, or targeted use. Only 50% of hospitals participated in all 6 quarters, which could have biased the results towards hospitals contributing more data; however, there was not a significant difference in hospital size or the rate of antifungal prescribing between these 2 groups, and the number of hospitals that contributed data was similar across all 6 quarters.

Understanding the characteristics of antifungal prescribing is important to design and implement effective antifungal stewardship efforts. Our study highlights important patterns of antifungal utilization in children. Most patients with an antifungal prescription had an oncologic-BMT diagnosis or prematurity, and antifungal use was minimal outside the hematology-oncology unit, NICU, and PICU. Currently, the main indication for antifungal use is prophylaxis in high-risk groups. Despite available clinical consensus guidelines, significant variability in antifungal prescribing was observed, which suggests the need for a standardized approach to antifungal prophylaxis in children. Based on these data, ASP programs may consider focusing their efforts on these high-risk populations and high-utilization units, with enhanced monitoring and optimization of prophylactic antifungal use.

## Supplementary material

For supplementary material accompanying this paper visit https://doi.org/10.1017/ash.2022.338. click here to view supplementary material
