# Supplementary material for: Characteristics of antifungal utilization for hospitalized children in the United States
Source: Antimicrob Steward Healthc Epidemiol. 2022 Dec 2;2(1):e190. doi: 10.1017/ash.2022.338 (PMC9726632; doi:10.1017/ash.2022.338)
Supplement: Supplementary file 1 [file S2732494X22003382sup001.docx]

**Supplemental Table 1: Classification of indications for antifungal use for data analysis**

| **Indications on data collection forms** | | **Study classification** |
| --- | --- | --- |
| **Indication for treatment** | **Empiric vs targeted** |  |
| Prophylaxis | N/A | Prophylaxis |
| Other indications:   - Community acquired infection - Healthcare-associated infection | - Empiric without culture(s) performed - Empiric but culture and susceptibility testing pending - Empiric without positive cultures | Empiric |
|  | - Empiric and pathogen identified - Targeted and pathogen and antimicrobial resistance confirmed | Targeted |
| Unknown | N/A | Unknown |

N/A: not applicable

**Supplemental Table 2: Azole antifungals by route of administration**

| **Azole antifungal** | **Parenteral,**  **(N=624)**  **No.** | **Enteral,**  **(N=864)**  **No.** | **Unknown,**  **(N=5)**  **No.** |
| --- | --- | --- | --- |
| Fluconazole | 513 | 535 | 3 |
| Voriconazole | 86 | 241 | 2 |
| Posaconazole | 24 | 68 | - |
| Itraconazole | - | 19 | - |
| Isavuconazole | 1 | - | - |
| Ketoconazole | - | 1 | - |
